# Overview of the Special Issue “Protein-Based Infection, Inheritance, and Memory”

**DOI:** 10.3390/ijms241411280

**Published:** 2023-07-10

**Authors:** Yury O. Chernoff, Anton A. Nizhnikov

**Affiliations:** 1 School of Biological Sciences, Georgia Institute of Technology, Krone EBB, 950 Atlantic Drive NW, Atlanta, GA 30332-2000, USA; 2 Laboratory for Proteomics of Supra-Organismal Systems, All-Russian Research Institute for Agricultural Microbiology, 3 Podbelskogo Sh., Pushkin, 196608 St. Petersburg, Russia; 3 Faculty of Biology, St. Petersburg State University, 7/9 Universitetskaya Emb., 199034 St. Petersburg, Russia

The Special Issue “Protein-Based Infection, Inheritance, and Memory” includes a set of experimental and review papers covering different aspects of protein memory, infection, and inheritance. The traditional view of biology separates nucleic acids as carriers of coded information from proteins as working macromolecules whose properties are encoded in this information but which are not capable of transmitting information by themselves. However, overwhelming evidence, mostly accumulated relatively recently, challenges this simplistic picture. Indeed, it has been demonstrated that the templated proliferation of protein isoforms can result in the reproduction and amplification of information encoded in the protein structure [1,2]. Therefore, self-perpetuating protein isoforms can become carriers of biological information that are not directly encoded in DNA sequences. While initial indications to the existence of non-DNA inheritance have been accumulating for years, with the most striking example of cortical inheritance in ciliates [3], specific mechanisms have emerged from studying transmissible protein isoforms (prions) [1,4]. Prions were initially described as infectious agents causing devastating diseases in humans (for example, kuru and Creutzfeldt–Jakob diseases) and other mammals (such as sheep scrapie, bovine spongiform encephalopathy or “mad cow” disease, and chronic wasting disease of cervids) [2,5]. Then, it was reported that prions, represented by different proteins but based on the same molecular mechanism, manifest themselves as heritable elements, transmitted via cytoplasm in yeast and other fungi [1,6]. In many cases, prions are fibrous cross-β aggregates (amyloids) that reproduce themselves and proliferate via the process of nucleated polymerization [7]. This same mechanism is also applicable to amyloids either involved in a variety of human diseases, such as the age-dependent, widespread, and incurable Alzheimer’s disease, as well as Parkinson’s disease and Amyotrophic Lateral Sclerosis (ALS) [8,9], or playing vital biological roles in archaea, bacteria, fungi, plants, and animals, including humans [10,11,12,13]. While some of cases of amyloid-associated diseases are heritable (that is, derived from DNA mutations), the majority of cases are sporadic, and prion-like transmissibility of protein assemblies has been observed at a cellular level and, in some experimental models, even at the organismal level [14,15,16]. This shows that naturally infectious or heritable prions represent only the tip of the iceberg, while similar processes are involved in a variety of biological and pathological events. Notably, prion-like behavior is not restricted to amyloids and other molecular mechanisms capable of transmitting protein-based information have also been uncovered [17,18]. Self-perpetuating protein isoforms that are similar to prions but not heritable through cell division have also been implicated in cellular memory. Such non-heritable molecular memory devices found in yeast have been termed mnemons [19]. Metastable prions may also maintain the cellular memory persisting in a small fraction of cells [20]. It appears that the retention of long-term memory in higher eukaryotes may include similar molecular mechanisms [21]. The ability of proteins to serve as information templates challenges existing biological paradigms and opens additional pathways for information transfer in biological systems, potentially playing a role in adaptation and evolution. The importance of this mechanism is still grossly underestimated by the majority of researchers [4]. The current issue covers molecular mechanisms controlling the ability of proteins to serve as information carriers in infection, inheritance, and memory.

The experimental paper by Kachkin et al. [22] describes the identification of a new human protein with amyloid properties, RAD51, that is a central player in DNA recombination and repair. While RAD51 is known to form a functional filamentous structure, this work points to its ability to produce typical cross-β amyloid fibrils as well. Such an ability was confirmed both for purified protein and in the *E. coli*-based C-DAG system, when a respective protein is accumulated on the surface of bacterial cells. The amyloid properties of RAD51 imply that aberrant protein-based assemblies could potentially affect DNA lesions, thus providing a possible link between protein-based and DNA-based transmission machineries.

The paper by Sulatskaya et al. [23] reviews the involvement of β-barrel proteins in amyloid formation in different organisms. Amyloids and β-barrels represent two widespread types of β-folds, and recent findings demonstrated that β-barrel proteins from different organisms including bacteria, plants and animals form amyloids that could be either functional or pathogenic like human SOD1’s amyloid state, which is associated with the development of ALS. The authors highlight the biological functions of amyloids formed by proteins containing β-barrel domains and discuss probable pathways of amyloid formation by such proteins.

The paper by Heumüller et al. [24] reviews the propagation and dissemination strategies of infectious mammalian prions. These prions are based on PrP protein and cause transmissible spongiform encephalopathies. Despite the well-demonstrated infectious nature of PrP-based agents, both sites of prion formation and pathways involved in prion transmission remain understudied and, to a certain extent, controversial. The authors specifically emphasize data derived from ex vivo cell models, explore the roles of extracellular vesicles and tunneling nanotubes, and address the link between viral and prion infections. 

The paper by Fedotov et al. [25] reviews approaches to noninvasive diagnostics of renal amyloidosis in humans. While not shown to be infectious between organisms, renal amyloidosis is based on the same molecular processes that underlie the transmission of amyloid-based prions. The establishment of noninvasive diagnostic approaches is crucial for early diagnosis and successful treatment of renal amyloidosis. The review critically assesses existing diagnostic techniques and explains how the principles and procedures derived from studying transmissible amyloids could be applied to clinical and pre-clinical detection of amyloids involved in other diseases, such as renal amyloids. 

The experimental paper by Akhtar et al. [26] addresses the mechanisms underlying the differences between transmissible prions acting as heritable elements, and non-transmissible mnemons, acting as carriers of cellular memory in yeast. The authors implicate the association between the endoplasmic reticulum membrane and the septin-based diffusion barrier between mother and daughter cells as major factors, preventing transmission of a mnemon from mothers to daughters. They also show that this barrier could sometimes be overcome in the first cell division after the mnemon formation, indicating that the difference between prions and mnemons is not absolute.

While these papers understandably do not cover the whole field of protein-based information transfer, they provide excellent examples of recent achievements and mark several areas of rapid growth that would undoubtedly produce further important results in the years to come. As such, we believe this Special Issue of *IJMS* would be of significant interest for readers. 
